# Idiosyncratic Profile of Perceived Emotional Intelligence and Post-Traumatic Growth in Breast Cancer Survivors: Findings of a Multiple Mediation Model

**DOI:** 10.3390/ijerph19148592

**Published:** 2022-07-14

**Authors:** Rocío Guil, Paula Ruiz-González, Lucía Morales-Sánchez, Rocío Gómez-Molinero, Paloma Gil-Olarte

**Affiliations:** 1 Department of Psychology, University of Cádiz, 11519 Cádiz, Spain; rocio.guil@uca.es (R.G.); lucia.morales@uca.es (L.M.-S.); rocio.gomez@uca.es (R.G.-M.); 2 Institute for Research and Innovation in Biomedical Sciences of Cádiz (INIBICA), 11009 Cádiz, Spain; 3 University Research Institute for Sustainable Social Development (INDESS), University of Cádiz, 11406 Cádiz, Spain

**Keywords:** breast cancer survivors, post-traumatic growth, perceived emotional intelligence, emotional attention, emotional clarity, emotional repair

## Abstract

Psycho-oncology research suggests that positive personal changes can occur after experiencing breast cancer. These changes can be understood as post-traumatic growth (PTG) and seem to be determined by emotional self-efficacy perception. This study aims to investigate the existence of different profiles of PTG and perceived emotional intelligence (PEI) among breast cancer survivors (BCSs) and healthy controls. Moreover, it aims to study the mechanisms through which PEI may mediate the relationship between disease survival and PTG. The total sample was 636 women divided into two groups: 56 BCS and 580 healthy controls who completed TMMS-24 and PTGI. The results displayed that BCSs apparently show a different profile of PTG and PEI compared to healthy women. The mediation analyses showed that survivorship explained 1.9% of PTG, increasing to 26.5% by the effect of PEI. An indirect effect showed that cancer survival predicts reduced levels of emotional attention, decreasing PTG. However, the most statistical indirect effect evidenced that BCSs regulate their emotions appropriately, having a powerful effect on PTG and counteracting the negative effects of poor emotional attention. Knowing the implications of PEI on PTG could improve follow-up from the time of diagnosis and supporting the patient to cope with the sequelae of the disease.

## 1. Introduction

Breast cancer diagnosis followed by the administered medical treatments may have an important negative impact on women’s physical and mental health. Several studies have demonstrated that breast cancer is associated with negative psychological reactions [1,2] and decreased well-being and quality of life [3,4]. Moreover, this life challenge is recognized as a traumatic life event that threatens the patient’s life, increasing psychological and psychiatric comorbidities [5,6]. However, despite the traumatic nature of breast cancer, in recent years, psycho-oncology research has increasingly focused on the positive changes that may arise as a result of coping with the illness [7,8]. These perceived positive outcomes are described as post-traumatic growth (PTG).

Tedeschi and Calhoun [9] defined PTG as a positive psychological change experienced as a result of adversity or other challenges to reach a higher level of life functioning. Growth refers to the personal development that goes beyond coping, meaning a qualitative change in people’s ability to function and adapt to adverse circumstances [10,11]. It would include a greater appreciation of life; a changed sense of priorities; deeper relationships; a greater sense of personal strength; recognition of new possibilities or pathways for one’s life; and spiritual development [9].

Considering that breast cancer diagnosis is a potentially life-threatening event, this disease can also be an opportunity for personal growth and social enrichment [12,13,14]. In this sense, many studies have evidenced that PTG is common among breast cancer survivors, even indicating a prevalence of 98% between 1 and 5.5 years, and suggesting its association with both psychological adjustment and psychological health [15,16,17,18]. Moreover, in a cross-sectional study developed by Sharma and Zhang [19], 80% of the 120 breast cancer patients evaluated were found to have moderate to high levels of PTG. More recently, a longitudinal investigation has shown that more than 97% of the 71 samples of breast cancer patients experienced PTG [20], suggesting that coping with this traumatic situation may facilitate PTG and future disease adjustment [21,22,23]. These results show that cognitive processing may facilitate the finding of positive meaning derived from the disease [24,25].

People, regardless of having suffered breast cancer, experience traumatic situations throughout the life cycle, such as dismissals, divorces, or deaths of loved ones. As in breast cancer patients, healthy individuals may have a hidden underlying trauma from these adverse circumstances that influences their ability to react adaptively and manifest a greater vulnerability to mental disorders. However, in line with Tedeschi and Calhoun [9], others may also emerge stronger and use past experiences for better coping [26].

In this sense, Tedeschi and Calhoun [9] identified that when a stressful event or trauma arises, the individual engages in a cognitive–emotional process characterized by active rumination, aimed at searching for the meaning of the crisis and managing emotions. Thus, they include in their general model of PTG several variables that may influence the PTG response. Among them are self-efficacy and the emotional and cognitive challenges produced by the traumatic event [27].

Regarding self-efficacy, greater confidence in one’s own abilities and skills is related to PTG [28,29]. Concerning emotional and cognitive challenges, health-threatening situations can also cause emotional dysregulation affecting psychological states and mental health [30,31,32,33]. In this regard, breast cancer patients experience a wide range of emotional states from the time of diagnosis and throughout the illness stages and disease progression [34]. Especially after treatment, reactions of anger, stress, anxiety, depression, and fear of recurrence arise, particularly in those women who have not overcome grief [35,36]. Therefore, it does not represent a punctual stressful event but involves a succession of unforeseen or unknown situations that are prolonged over a wide period, causing emotional states that are difficult to manage. In line with Tedeschi and Calhoun [27], it is expected that self-efficacy regarding emotional-state management will be a facilitating factor of PTG, with this capacity being implicit in the concept of perceived emotional intelligence (PEI).

The concept of emotional intelligence (EI) emerged in the early 1990s as a construct that includes aspects such as empathy, emotional expression, self-control, and problem solving, among others. Traditionally, EI has been highlighted as a personal resource that enhances psychological adjustment and personal transformation after facing adversities [37,38,39,40]. EI has been operationalized in two ways: (1) as an ability similar to general intelligence and (2) as a trait.

Understood as an ability, Mayer and Salovey [41] define EI under their capabilities model as a type of social intelligence with four main characteristics: “the ability to perceive accurately, appraise, and express emotion; the ability to access and/or generate feelings when they facilitate thought; the ability to understand emotion and emotional knowledge; and the ability to regulate emotions to promote emotional and intellectual growth” (p. 10).

On the other hand, other authors have conceptualized EI as a trait (mixed or trait models), in which EI is considered as a stable personality trait, behavioral tendency, and self-perceived ability [42]. This proposal was materialized through various scales such as the trait meta-mood scale (TMMS). The TMMS is a trait scale of meta-mood cognition, which refers to people’s perceptions of their own emotional abilities. The authors refer to this measure as PEI defined as beliefs in one’s abilities to attend, understand, and repair emotional states [43]. Specifically, it is a chain process, where the perceived ability to regulate moods seems to be determined by the ability to pay attention to emotions and perceive them clearly [44,45,46].

Research suggests that emotional intelligence assessed as perceived abilities with self-report measures is more strongly related to mental and psychological health than it is assessed through ability measures [47].

Thus, perceived emotional self-efficacy has been shown to have positive outcomes on psychological health, enhancing adaptation to illness [48,49], as well as cognitive processing that drives emotional, intellectual, and personal growth following a struggle process [50,51,52]. That is, perception, emotional expressiveness, and emotional coping are positively related to a sense of greater personal strength, as well as life priority changes after trauma [50,53,54,55]. In this line, Mohanty et al. [56] found that emotional regulation was a strong predictor of PTG after facing impactful life changes in undergraduate students. More recently, Kira et al. [57] found that an individual’s innate will to exist, live, and survive promoted PTG through the indirect effect of emotional regulation.

This process also applies to breast cancer survivors, where the literature has shown that emotional intelligence exerts a direct influence on PTG levels after survival [58]. In this sense, Pat-Horenczyk et al. [59] conducted a psychological intervention with breast cancer survivors, showing that the experimental group that received training in emotional regulation reported an increase in PTG levels. Thus, the ability of breast cancer survivors to withstand life crises, such as the effects of medical treatments, seems to be enhanced by the direct and mediated effect of cognitive–emotional regulation [13,60,61]. However, not all PEI dimensions seem to act in the same way. Specifically, low or high levels of emotional attention lead to collateral effects [62] if it is not accompanied by an adequate ability to discriminate emotions and repair them effectively [63].

To our knowledge, non-studies have focused on the relationship between breast cancer survivorship, PEI, and PTG. Therefore, understanding how PEI contributes to promoting PTG seems of utmost importance to improve a better quality of life and psychological state after medical treatment for breast cancer.

Hence, the first aim of this research is to examine the existence of different clusters between breast cancer survivors and a group of healthy controls concerning PTG and PEI, as well as to examine the elements in which they differ in a statistically significant way. That is, to check if there are differences in the way of growing and managing emotions among women who, in addition to having experienced common adverse situations as healthy controls, are in the particular situation of having suffered from breast cancer.

Building on our previous research, where we found differences in PEI dimensions between breast cancer survivors and healthy controls, we hypothesize that the range of emotional responses that breast cancer survivors experience can be reflected in an idiosyncratic profile in the management of such states that allow them to emerge stronger from this situation.

The second objective is to analyze the possible differences in the explanatory and predictive ability of the PEI on PTG in the different clusters. In line with this objective, PEI is expected to influence the development of PTG in both breast cancer survivors and healthy controls. Finally, the third objective is to know the processes through which the dimensions of the PEI (attention, clarity, and emotional repair) could influence, in a direct and/or mediated way on PTG. It is hypothesized that women with breast cancer who show adequate emotional clarity and repair will have increased PTG levels. Conversely, low or high levels of emotional attention would act as a risk factor, reducing personal growth after the disease.

## 2. Materials and Methods

### 2.1. Sample

The study participants were 636 women divided into two groups. Group 1 consisted of 56 breast cancer survivors (M_age_ = 51.77; *SD* = 8.92). The control group included 580 disease-free women (M_age_ = 40.40; *SD* = 9.71). The difference in the size of the groups is due to the proportional distribution of the incidence of breast cancer in the Spanish female population (1 woman with breast cancer for every 8–10 healthy women).

Considering some sociodemographic characteristics of the participants, such as marital status, we found that in Group 1, 69.7% were married, 10.7% were divorced, 8.9% were single, 7.1% were common-law partners, and 3.6% were widowed. In terms of employment status, 32.1% were pensioners, 19.6% were housewives, 17.9% were on sick leave, 17.9% were unemployed, 7.1% were employed, and 5.4% were self-employed. Concerning educational level, 35.7% had university studies, 30.4% had primary studies, 17.9% had vocational training, 12.5% had secondary studies, and 3.5% had no studies.

In Group 2, in terms of marital status, 39.6% were married, 37.9% were single, 12.2% were common-law partners, 9.3% were divorced, and 1% were widowed. Attending employment status, 60.7% were self-employed, 17.1% were unemployed, 12.6% were employed, 4.8% were housewives, 2.9% were pensioners, and 1.9% were on sick leave. According to educational level, 74.9% had university studies, 12.9% had vocational training, 11% had secondary studies, and 1.2% had primary studies.

The common inclusion criteria of both groups were: (i) being a woman; (ii) to being older than 18 years; (iii) having a reading and writing level that made it possible to understand the different tests; (iv) not being, at the time of the study, under psychiatric and/or psychological treatment due to a serious mental disorder; (v) not being under psychoactive medication at the time of the interview; (vi) not presenting any serious or incapacitating pathology. Moreover, in Group 1, it was also taken into account: (i) having received a breast cancer diagnosis; (ii) to have been diagnosed with breast cancer at least 1 year before participating in the study or having received the medical discharge; and (iii) not being diagnosed with other serious pathologies concomitant with the oncological process.

### 2.2. Procedure

A cross-sectional, analytical, and ex post facto design was used to measure and detect the relationships between the variables collected. Women in group 1 who met the inclusion criteria were randomly recruited from the oncology units of the reference hospitals of the province of Cadiz, Spain. Women in the control group (Group 2) were randomly selected from a group of women from the province of Cadiz through social networks and the general population. All participants completed the questionnaires individually through digital devices. Participation was voluntary and they received no financial compensation for participation in the study. Before participation, they had to sign an informed consent. The study approval was obtained from the Ethics Committee of the referral hospitals (project identification code: PIN0109-2018).

### 2.3. Measures and Instruments

*Trait Emotional Intelligence*. The trait meta-mood scale-24 ((TMMS-24); Salovey et al. [42]; Spanish version by Fernández-Berrocal et al. [64]), is a self-reported instrument that evaluates the ability to be aware of our emotions and our regulation competency. This inventory consists of 24 items that assess three important dimensions of PEI: emotional attention, which refers to people’s self-perception of their ability to pay attention to their moods and emotions; emotional clarity, which is defined as the self-perception of people’s ability to discriminate their emotions; and emotional repair, which refers to the self-perception of people’s ability to repair and manage their emotional states. It is answered using a 5-point Likert scale, with options ranging from 1 (strongly disagree) to 5 (strongly agree). For emotional clarity and emotional repair, it is considered that higher levels correspond to higher excellence. In turn, emotional attention is considered inadequate at both, high and low levels. Cronbach’s alpha for each of the subscales in the Spanish version were 0.90, 0.90, and 0.86, respectively [60].

*Post-traumatic Growth*. Post-traumatic growth inventory ((PTGI); Tedeschi & Calhoun, [65]; Spanish version by Weiss & Berger [66]). The PTGI evaluates the perception of positive life changes following a traumatic event, and it consists of five domains: relating to others, new possibilities, personal strength, spiritual change, and appreciation of life. The 21 items are rated on a 5-point Likert scale, ranging from 0 (I did not experience this change as a result of my crisis) to 5 (I experienced this change to a very great degree as a result of my crisis). The scores range from 0 to 105, and higher scores indicate that a person perceived the development of greater PTG resulting from their traumatic experience. We analyzed the global PTG score (sum of all of the items). The PTGI has shown excellent internal consistency for the total scale (α = 0.90) in the Spanish version [66].

### 2.4. Statistical Analysis

Preliminary analyses were performed to obtain descriptive statistics and internal consistencies of all measures were assessed through Cronbach’s alpha. Moreover, a two-step cluster was carried out to find different profiles in terms of age, levels of PTG, and PEI according to being or not a breast cancer survivor. A one-way analysis of variance (ANOVA) was performed using Scheffé’s post hoc test to compare groups and determine significant differences between them according to the indicated variables. To check the effect size of the ANOVA, it was calculated through Cohen’s d, considering as follows: small effect (values between 0.20 and 0.30), medium effect (around 0.50 and 0.80), and large effect (more than 0.80). Moreover, linear regression analyses were carried out in each group to examine the explanatory and predictive ability of these variables.

All of these analyses were carried out with the SPSS (version 20.0; IBM, Chicago, IL, USA). Finally, a serial multiple mediation analysis was performed using Model 6 in the PROCESS macro tool. Moreover, a bootstrapping resampling method of 10.000 simulations was used to obtain 95% confidence intervals and, thus, assess the significance of the mediating effects. To verify which indirect effect had more statistical weight, we performed specific contrasts for indirect effects.

Concerning the mediation analysis, breast cancer survivorship was considered the first variable (predictor, X), and PTG was determined as the outcome variable (Y). Additionally, emotional attention (M1), emotional clarity (M2), and emotional repair (M3) were considered the mediator variables. In the serial mediation analysis, the mediators have a direct effect on each other and it is assumed that the independent variable (in this case, breast cancer survivorship) affects the mediators in a serial manner, lastly influencing the dependent variables; that is PTG in the present study.

**Figure 1:**
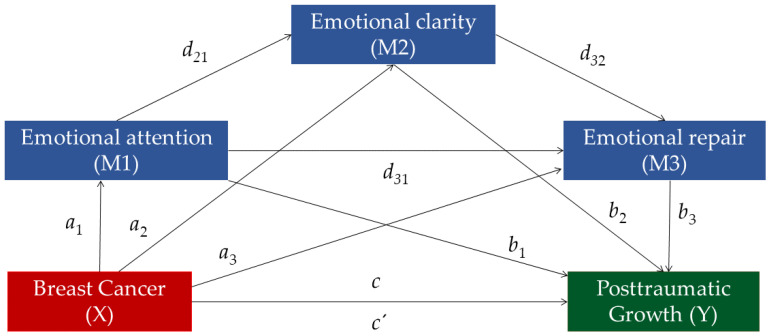
Serial Multiple Mediation Analysis in a statistical diagram with the three mediators (Emotional Attention, Emotional Clarity, and Emotional Repair).

Figure 1 illustrates the relations between the variables in the multiple serial mediation model. The total effect (c) refers to the relationship between breast cancer survival and PTG, without accounting for the mediators, while the direct effect (c’) refers to that relationship after controlling for all the mediators. The total indirect effect (a) represents the association between the predictor variable and the three mediators (a_1_, a_2_, and a_3_), and the total indirect effect (b) refers to the effect of the three mediators in the relationship between independent and dependent variables (b_1_, b_2_, and b_3_). Finally, the total indirect effect (d) refers to the relationships between all three mediators (d_21_, d_31_, and d_32_), and the specific indirect effect (a_3_b_3_) refers to the role of a specific mediator in the relationship between breast cancer survival and PTG levels.

## 3. Results

Table 1 displays reliability coefficients (Cronbach’s alpha) and descriptive statistics (means and standard deviation) between all the study variables for the total sample and women in both groups separately. Internal consistencies of all instruments administered were above 0.93. Descriptive statistics indicate that breast cancer survivors and healthy controls showed adequate levels in all dimensions of PEI as well as good PTG.

A two-step cluster analysis was conducted to explore possible profiles according to the breast cancer survivor or nonsurvivor status (categorical variable). PTG and the three dimensions of PEI (continuous variables) were introduced as proxies for cluster attributes. Moreover, age was incorporated because of its association with cancer development, as appears to be reflected in the descriptive statistics.

**Figure 2:**
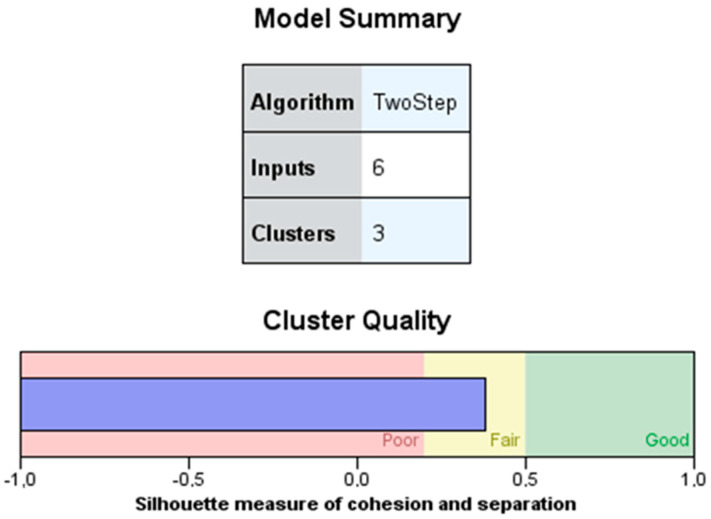
Model summary and cluster quality graph.

The resulting model indicated the formation of three clusters. The cluster quality plot indicated that the result is correct (average silhouette = 0.4) (Figure 2).

**Figure 3:**
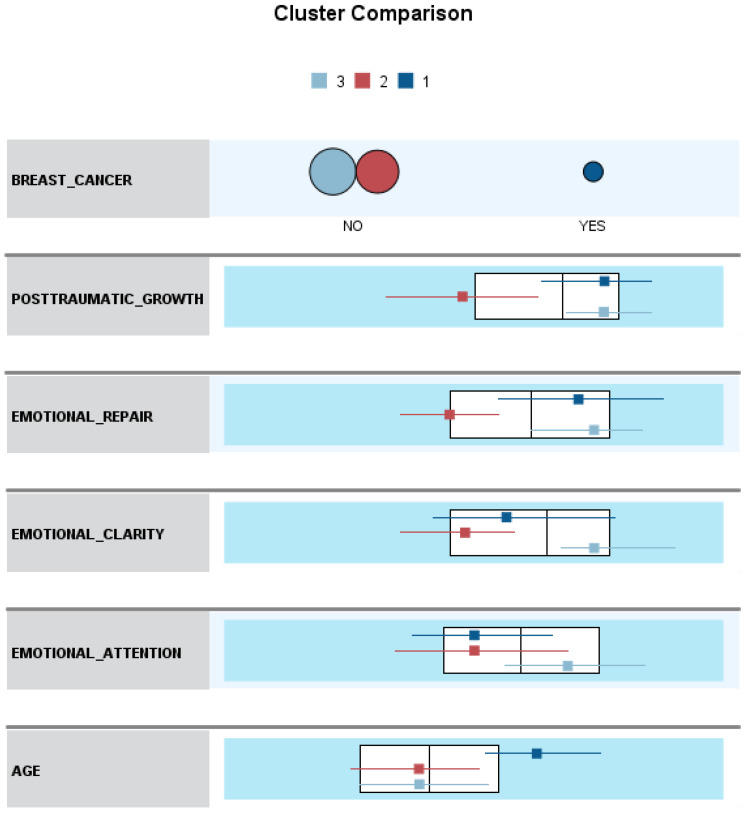
Comparison of clusters concerning breast cancer survivor and nonsurvivor status.

Figure 3 illustrates the variables evaluated in the rows and the clusters in the columns, showing three distinct profiles:

Cluster 1 (N = 56; 8.8%). Older (M = 51.77) female breast cancer survivors with high levels of PTG (M = 77.09) and emotional repair (M = 30.32) and low levels of emotional attention (M = 24.50) and emotional clarity (M = 27.14).

Cluster 2 (N = 267; 42.0%). Healthy, younger women (M = 40.03) with low levels of PTG (M = 49.26), emotional attention (M = 24.67), emotional clarity (M = 24.05), and emotional repair (M = 23.18).

Cluster 3 (N = 313; 49.2%). Healthy, younger women (M = 40.72) with high levels of PTG (M = 80.11), emotional attention (M = 29.58), emotional clarity (M = 32.65), and emotional repair (M = 31.72).

To determine whether the differences observed between the three groups were statistically significant, one-way ANOVA analyses were performed. We found statistically significant differences between age (F (2,633) = 35.81, *p* = 0.000), PTG (F (2,633) = 247.40; *p* = 0.000), emotional attention (F (2,633) = 41.81; *p* = 0.000), emotional clarity (F (2,633) = 182.95, *p* = 0.000), and emotional repair levels (F (2,633) = 199.47, *p* = 0.000).

A post hoc pairwise multiple comparisons was carried out using the Scheffé test to identify which groups had statistically significant different means. Post hoc comparisons indicated that:

(1) Breast cancer survivors (cluster 1) differed significantly in age (*p* = 0.000) and showed higher levels of PTG (*p* = 0.000), emotional clarity (*p* = 0.001), and emotional repair (*p* = 0.000) compared to healthy women with low PTG (cluster 2). Cohen’s *d* results showed a strong effect for age (*d* = 1.25; [8.26–15.22] 95% CI), PTG (*d* = 1.44; [21.68–33.96] 95% CI), and emotional repair (*d* = 1.13; [5.26–9.02] 95% CI), and a medium effect for emotional clarity (*d* = 0.47; [1.13–5.05] 95% CI). In emotional attention, no significant differences were found between groups (*p* > 0.05).
(2) The mean scores of breast cancer survivors (cluster 1) differed significantly from healthy women with high PTG (cluster 3) in being older (*p* = 0.000) and showing lower levels of emotional attention (*p* = 0.000) and emotional clarity (*p* = 0.000). Cohen’s d results demonstrated a strong effect for age (*d* = 1.19; [7.62–14.49] 95% CI) and emotional clarity (*d* = 0.91; [3.57–7.43] 95% CI), and medium effect for emotional attention (*d* = 0.76; [2.65–7.51] 95% CI). No differences were found in PTG levels and emotional repair (*p* > 0.05).
(3) Healthy women with low levels of PTG (cluster 2) differed significantly from women with high PTG levels (cluster 3) in lower scores on PTG (*p* = 0.000) and emotional attention (*p* = 0.000), clarity (*p* = 0.000), and emotional repair (*p* = 0.000). Cohen’s *d* results showed a strong effect for PTG (*d* = 1.78; [27.36,34.33] 95% CI), emotional clarity (*d* = 1.62; [7.49–9.70] 95% CI), and emotional repair (*d* = 1.70; [7.47–9.60] 95% CI), and medium effect for emotional attention (*d* = 0.71; [3.52–6.30] 95% CI).

It is confirmed that in our sample, women who have survived breast cancer were older than women in the control group. In addition, significantly different profiles of PEI were found between breast cancer survivors and healthy controls, as well as between healthy controls with high and low PTG.

Since these analyses did not confirm the existence of a causal relationship between the variables, we performed three linear regression analyses to test the explanatory and predictive ability of the condition of having suffered cancer, age, and the dimensions of PEI on PTG.

The first regression analysis was applied to the total sample, including cancer survival, age, and the three dimensions of PEI as predictor variables. The second and third regression analyses were carried out only on healthy controls or breast cancer survivors, respectively. In both analyses, the same predictor variables were included except cancer or noncancer status.

Table 2 shows the results of the three regression analyses performed, showing only the statistically significant ones.

As can be observed, when analyzing the total sample, breast cancer survival and PEI were significant predictors of PTG. Likewise, emotional attention was the variable that explained and predicted the levels of PTG in breast cancer survivors. For healthy controls, emotional attention and emotional repair showed explanatory and predictive ability on the dependent variable.

In all three analyses, all variables positively predicted PTG except age, which showed no predictive ability.

Given the profiles found and the influence that PEI seems to exert on both groups, a mediation analysis was carried out. This analysis aimed to determine the mechanisms by which PEI mediates the relationship between breast cancer survivorship and PTG. Table 3 displays the significant direct and indirect effects.

**Figure 4:**
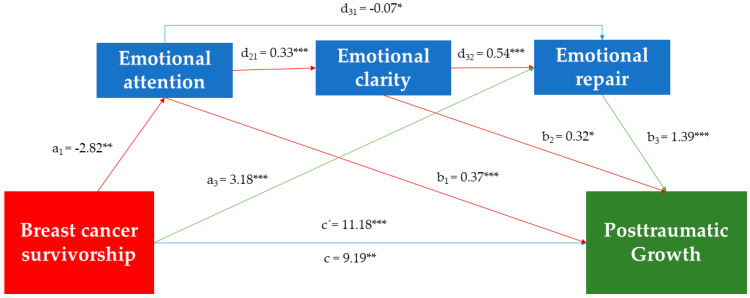
Direct and indirect effects for mediation model. Notes. * *p* < 0.05; ** *p* < 0.01; *** *p* < 0.001.

The mediating path model with path coefficients is summarized in Figure 4.

The serial mediation analysis indicated a partial mediation model. The total amount of variance explained by the global mediation model was 26.5% (R^2^ = 0.265; *p* = 0.000). The analysis revealed that breast cancer survivorship explained only 1.9% (R^2^ = 0.019; c: β = 9.19; *p* = 0.001) of the variance in PTG. Thus, 24.5% of the variance in PTG in both groups was attributed to direct or indirect effects of the emotional attention, clarity, and repair (R^2^ = 0.246; c’: β = 11.18; *p* = 0.000).

Considering the statistically significant direct effects, we found a positive predictive association between breast cancer survivorship and emotional repair (a_3_: β = 3.18; *p =* 0.000). Moreover, the three dimensions of PEI (emotional attention, emotional clarity, and emotional repair) were directly associated with PTG regardless of having survived breast cancer (b_1_: β = 0.37, *p* = 0.001; b_2_: β = 0.32; *p* = 0.03; b_3_: β = 1.39, *p* = 0.000, respectively). Likewise, emotional attention was positively related to emotional clarity (d_21_: β = 0.33; *p =* 0.000) and emotional clarity to mood repair (d_32_: β = 0.54; *p* = 0.000). Other significant direct effects indicated a negative association between breast cancer survivorship and emotional attention (a_1_: β = −2.82; *p* = 0.005), and between emotional attention and emotional repair (d_31_: β = −0.07; *p =* 0.046).

According to the regression coefficients and considering that CI (95%) did not include zero, we obtained four specific indirect effects: three contribute to decreasing the levels of PTG and one increased it.

Concerning the negative indirect effects, indirect effect 1 (a_1_b_1_) revealed that breast cancer survivorship decreased emotional attention levels, and in turn, PTG (β = −1.03; BootSE = 0.50; 95% BootCI = −2.13, −0.20). Moreover, indirect effect 4 (a_1_d_21_b_2_) indicated that breast cancer survival was associated with low levels of emotional attention, which is related to a decrease in the ability to discriminate emotions, and in turn, with low PTG (β = −0.30; BootSE = 0.19; 95% BootCI = −0.73, −0.01). Finally, indirect effect 7 (a_1_d_21_d_32_b_3_) showed that breast cancer survivorship was associated with lower levels of emotional attention, which in turn was linked to lower emotional clarity and to lower levels of repair, affecting the development of PTG (β = −0.70; BootSE = 0.28; 95% BootCI = 0.1.31, −0.21).

Regarding the positive indirect effect, indirect effect 3 (a_3_b_3_) revealed that breast cancer survivorship was associated with higher emotional repair and, consequently, with greater personal growth after adversity (β = 4.41; BootSE = 1.31; 95% BootCI = 1.96, 7.14).

To determine which indirect effect had the greatest statistical weight, an analysis of contrasts was carried out. Table 3 shows the statistically significant contrasts with a 95% confidence interval. Considering the sign of the coefficients, the analyses showed that indirect effect 3 had greater statistical weight, that is, the mediated relationship of emotional repair between breast cancer survival and PTG. Thus, breast cancer survivors tend to show a greater ability to regulate negative emotions, and in turn, higher levels of PTG.

## 4. Discussion

This research aimed to explore the levels of PTG and PEI in breast cancer survivors, as well as the existence of an idiosyncratic profile in these variables compared to healthy controls. Although there are studies demonstrating the influence of PEI on PTG [37,48], this is the first study to point to the existence of a characteristic profile in this growing oncology population, as well as the exact mechanism through which PEI mediates the relationship between surviving breast cancer and PTG.

Agreeing with the evidence that reflects that the diagnosis of chronic diseases has a negative psychological impact on patients [1,4], the descriptive statistics showed adequate levels of PTG and PEI dimensions (emotional attention, clarity, and repair) in both groups, breast cancer survivors and healthy controls. These findings are in line with studies that focus on the positive changes that occur after experiencing traumatic events [7,8] and show that this population can emerge stronger, developing moderate–high levels of PTG after coping with the disease diagnosis and adjuvant treatments [14,16,20]. Moreover, these results are in line with previous research indicating that breast cancer survivors manage their emotions appropriately [60].

Regarding the two-stage cluster analysis, we confirmed the existence of three differentiated group profiles: one formed by breast cancer survivors with higher levels of PTG and emotional repair, and lower levels of emotional attention and clarity. On the other hand, two groups of healthy controls differed in showing high or low levels of PTG and PEI dimensions.

The finding of two distinct clusters of healthy women confirms those studies that indicate that some people will be affected in their normal functioning, while others will be strengthened by experiencing adverse circumstances [26]. Likewise, the way emotions are managed differs among healthy groups.

Concerning breast cancer survivors, they reach high levels of PTG, as one of the clusters formed by healthy controls, suggesting that breast cancer could be an opportunity for personal growth [12,13]. However, they differ in terms of PEI dimensions as confirmed by the post hoc comparisons. On one hand, they are similar to the group of healthy women with high PTG in showing a high ability to repair emotions. This is in line with studies demonstrating that emotional regulation is a central factor in experiencing positive personal changes [13,56,61].

On the other hand, they resemble healthy controls with low PTG in low scores of emotional attention. This suggests that breast cancer survivors experience positive personal changes despite paying little attention to their emotions. It is apparently contradictory to studies that highlight the need to show adequate scores on all three dimensions of PEI for psychological adjustment [43]. In short, these results confirm our first hypothesis that breast cancer survivors develop an idiosyncratic PEI profile linked to PTG, resembling in some factors healthy women with high PTG, and in others, healthy controls with low PTG.

Regression analyses confirmed that both breast cancer survivorship and all dimensions of PEI explained and predicted PTG regardless of age. Hence, and similar to other studies, our results suggest that women who face this traumatic disease may emerge strengthened [19,21,22,23]. Moreover, these findings are also in line with studies demonstrating that emotional intelligence contributes to positive psychological and personal changes after adversity [37,38,51,52,55].

Our second hypothesis is confirmed due to the PEI dimensions predicting PTG in each of the study groups. In the case of women who have experienced this disease, emotional attention is the only variable that positively predicted PTG. This is in line with Tedeschi and Calhoun [9], who reflect on the importance of active cognitive–emotional rumination for growth and better psychological functioning after negative situations. This confirms the idiosyncratic profile previously found, where paying attention to emotions promotes personal change after adversity in the breast oncology population. Concerning healthy controls, who have likely experienced other types of traumatic situations, we find other predictive variables. In this case, it is emotional attention together with emotional repair which are the variables with positive predictive ability. That is, those women who, in addition to paying attention, perceive themselves to be self-effective in regulating negative emotions, will see increased levels of PTG regardless of their clinical situation. This is in line with findings indicating that adequate values on both variables are related to psychological adjustment [44]. These results confirm that breast cancer survivorship implies a specific traumatic situation that confers a differentiated profile to the patients.

Our serial mediation model showed that breast cancer survivorship explained 1.9% of the total PTG variance, increasing to 26.5% when PEI was incorporated. Regarding direct effects and in line with regression, it is confirmed that being a breast cancer survivor and having higher levels in all three dimensions of PEI lead to the development of greater PTG following life challenges [22,23,50,51]. Moreover, our direct effects revealed positive relationships between the three mediators; specifically, emotional attention leads to higher emotional clarity and the latter to higher emotional repair levels [43,45]. In this sense, the literature indicates that paying attention to emotions, perceiving them clearly, and repairing negative ones act as protective factors that improve psychological adjustment. Likewise, it is shown that these dimensions act in a serial chain, although with certain independence [60,63].

Regarding the direct effects of breast cancer on the different dimensions of PEI, we found that breast cancer survival has been linked to less attention to emotions, but a greater ability to effectively regulate negative emotions. These results are consistent with studies indicating that after breast cancer treatment, emotion regulation values can be even higher than healthy controls, suggesting that this traumatic experience may increase their perceived ability to modify negative emotional states [7,48,60].

Moreover, indirect effects show different results on PTG depending on PEI dimensions. Specifically, breast cancer survival predicts reduced levels of emotional attention, thus influencing a decrease in the development of PTG. This is in line with Davis and Nichols [62] who showed possible collateral effects in people who do not attend to their emotions. This negative outcome could also be produced by the mediated effect of attention on clarity and emotional repair, as well as by the serial relationship between the three dimensions, thus limiting the perception of positive changes after the experience of complicated situations. Hence, emotional attention seems to be the main variable that determines the difference concerning healthy controls in the development of personal growth after circumstances of struggle, because of its possible negative influence on PTG, as well as its effect on emotional clarity and repair.

However, the indirect effect with the most statistical weight evidenced that breast cancer survivors are more confident in their ability to regulate negative emotions, having a powerful effect on PTG levels [59] and counteracting the negative effects that poor emotional attention can have.

Our research highlighted emotional attention and emotional repair as determinant elements of our understanding of complex associations between breast cancer survivorship and PTG. Specifically, the lack of attention to emotions seems to play a determinant role as a risk factor in the experience of positive psychological changes after breast cancer treatments. However, these negative effects appear to be reversed because of their perceived high self-efficacy in emotional regulation [60], leading to greater personal growth. In this sense, the third hypothesis is partially confirmed, since emotional clarity was not linked to PTG after disease survival.

As a limitation of the study and despite applying the bootstrapping technique in the mediation analysis, we highlight the small sample size of breast cancer survivors. Thus, the interpretation of the results should be made with caution. Moreover, since it is a cross-sectional study, we cannot know whether the values obtained in the study variables are a consequence of the diagnosis or treatments or were prior to them. In other words, the causal interpretation is problematic because it is a cross-sectional study. Another limitation was the use of self-report measures that may cause responses to be biased. In this sense, it is proposed as future lines to combine it with objective measures of ability due to their high reliability and validity. In addition, it is proposed to confirm these results with studies in which the sample size of breast cancer survivors was larger or to carry out longitudinal studies to determine temporal changes and causal relationships between the perception of emotional management and personal growth. Finally, the study of medical variables could be included to determine whether the processes found are the same or may vary as a result of the specific characteristics of the diagnosis and treatments.

## 5. Conclusions

This study highlights the importance of maintaining adequate levels of emotional attention due to its apparent serial influence on emotion comprehension and repair of negative emotional states in both healthy women and breast cancer survivors. Specifically, in the latter, repairing negative emotions appears to exert a strong influence on personal growth after adversity.

Knowing the implications of PEI on personal growth in breast cancer survivors could improve patient follow-up from the time of diagnosis. In this sense, breast cancer rehabilitation should include not only the medical approach but also a comprehensive strategy that supports the patient for coping with the disease, treatments, and sequelae, allowing them to recover their functional status after the medical treatment. In short, optimizing the quality of care received and offering personalized attention would have a clinical and social impact. Specifically, it would improve psychological state and patient’s quality of life and reduce unnecessary care costs derived from psychological comorbidities in the general public health system. However, to establish the basis for designing interventions, it is necessary that studies be carried out in this field with larger samples and provide empirical evidence on the influence of emotional self-perceptions on PTG. It is also proposed to address their possible malleability as a preliminary step to designing and developing programs that promote PTG after breast cancer survivorship.

## Figures and Tables

**Table 1:** Internal Consistencies and descriptive statistics of the total sample, and for the breast cancer survivors and control group.

Variables		Total Sample		BC Survivors		Control Group	
	α	M	*SD*	M	*SD*	M	*SD*
Age	-	41.40	10.12	51.77	8.91	40.40	9.71
PTG	0.95	66.89	22.69	77.08	17.70	65.91	22.89
EA	0.92	27.07	7.24	24.50	7.04	27.32	7.21
EC	0.94	28.55	6.80	27.14	7.18	28.68	6.76
ER	0.91	28.01	6.64	30.32	7.16	27.78	6.55

Notes. α = Cronbach’s alpha; M = mean; *SD* = standard deviation; BC = breast cancer; PTG = post-traumatic growth; EA = emotional attention; EC = emotional clarity; ER = emotional repair.

**Table 2:** Summary of regression analyses.

Regression Analyses	Predictor Variables	R^2^	F	β	*p*
Total sample	Breast cancer	0.27	45.41 ***	9.49	0.001
	EA			0.36	0.003
	EC			0.32	0.026
	ER			1.39	0.000
Healthy controls	EA	0.27	53.09 ***	0.29	0.021
	ER			1.56	0.000
Breast cancer survivors	EA	0.23	3.83 **	0.84	0.036

Notes. EA = emotional attention; EC = emotional clarity; ER = emotional repair; R^2^ = coefficient of determination; F = F-Snedecor statistic; β = nonstandardized coefficients; *p* = *p*-value; ** *p* < 0.01; *** *p* < 0.001.

**Table 3:** Serial Multiple Mediator Model: Model summary and direct and indirect effects.

**Model Summary**	**R^2^**	**MSE**	**F**	**df1**	**df2**	***p* (sig)**	
	0.265	381.13	56.81	4.00	631.00	0.000	
**Total effect model** BCS on PTG	0.019	505.87	12.62	1.00	634.00	0.004	
					95% CI		
**Total effect**	**Path**	**Coeff.**	**SE**	***T***	***p***	**BootLL**	**BootUL**
BCS on PTG without accounting for mediators	c	9.19	2.78	3.30	0.001	3.72	14.66
**Direct Effects**							
BCS on PTG when accounting for mediators	c’	11.18	3.15	3.55	0.000	5.00	17.36
BCS on EA	a_1_	−2.82	1.01	−2.80	0.005	−4.80	−0.84
BCS on ER	a_3_	3.18	0.79	4.02	0.000	1.63	4.73
EA on PTG	b_1_	0.37	0.12	3.18	0.001	0.14	0.59
EC on PTG	b_2_	0.32	0.14	2.21	0.03	0.04	0.60
ER on PTG	b_3_	1.39	0.14	10.03	0.000	1.12	1.66
EA on EC	d_21_	0.33	0.04	9.51	0.000	0.26	0.40
EA on ER	d_31_	−0.07	0.03	−1.99	0.046	−0.13	−0.00
EC on ER	d_32_	0.54	0.04	15.35	0.000	0.47	0.61
**Indirect effects**							
Ind_1_. BCS on PTG via EA	a_1_b_1_	−1.03	0.50			−2.13	−0.20
Ind_3_. BCS on PTG via ER	a_3_b_3_	4.41	1.31			1.96	7.14
Ind_4_. BCS on PTG via EA and EC in serial	a_1_d_21_b_2_	−0.30	0.19			−0.73	−0.01
Ind_7_. BCS on PTG via EA, EC, and ER in serial	a_1_d_21_d_32_b_3_	−0.70	0.28			−1.31	−0.21
**Specific indirect effect contrast definitions**							
(C2) Ind_1_ minus Ind_3_		−5.44	1.48			−8.46	−2.67
(C4) Ind_1_ minus Ind_5_		−1.29	0.60			−2.61	−0.28
(C7) Ind_2_ minus Ind_3_		−4.60	1.32			−7.32	−2.12
(C12) Ind_3_ minus Ind_4_		4.71	1.33			2.21	7.45
(C13) Ind_3_ minus Ind_5_		4.15	1.32			1.69	6.91
(C14) Ind_3_ minus Ind_6_		4.86	1.46			2.20	7.87
(C15) Ind_3_ minus Ind_7_		5.11	1.42			2.48	8.06
(C16) Ind_4_ minus Ind_5_		−0.56	0.30			−1.26	−0.08
(C18) Ind_4_ minus Ind_7_		0.41	0.26			0.02	1.01
(C20) Ind_5_ minus Ind_7_		0.96	0.43			0.26	1.92

Notes. BCS = breast cancer survivorship; PTG = post-traumatic growth; Coeff. = nonstandardized B coefficients, SE = standard errors, CI = bias-corrected and accelerated 95% confidence interval, BootLL = lower limit, BootUL = upper limit. Model 6: Y = post-traumatic Growth; X = breast cancer survivorship; M1 = emotional attention (AE); M2 = emotional clarity (EC); M3 = emotional repair (ER). N = 636.

## Data Availability

Data provided upon request.
